# Washed microbiota transplantation effectively treats a case of acute severe ulcerative colitis combined with viral myocarditis

**DOI:** 10.7555/JBR.39.20250036

**Published:** 2025-05-20

**Authors:** Rujun Ai, Yan Jin, Faming Zhang, Bota Cui, Guozhong Ji

**Affiliations:** 1 Department of Microbiota Medicine & Medical Center for Digestive Diseases, the Second Affiliated Hospital of Nanjing Medical University, Nanjing, Jiangsu 210011, China; 2 Key Lab of Holistic Integrative Enterology, Nanjing Medical University, Nanjing, Jiangsu 211100, China; 3 Wuxi Second People's Hospital, Affiliated Middle School Hospital of Jiangnan University, Wuxi, Jiangsu 214000, China

**Keywords:** fecal microbiota transplant, microbiome, cytomegalovirus, Epstein-Barr virus, ulcerative colitis, viral myocarditis

## Abstract

Viral myocarditis is a rare but life-threatening complication in patients with ulcerative colitis. Management of myocarditis is primarily supportive, because there are currently no established targeted therapies. Recent studies have increasingly highlighted the association between the gut microbiota and myocarditis. Here, we report a case of acute severe ulcerative colitis complicated by cytomegalovirus and Epstein-Barr virus co-infections that led to viral myocarditis. The patient experienced rapid remission of both intestinal and cardiac symptoms following washed microbiota transplantation, suggesting this intervention may serve as a potential alternative treatment for these life-threatening conditions.

## Introduction

Myocarditis is a life-threatening complication in patients with inflammatory bowel disease^[1]^. It is primarily associated with viral infections, such as enteroviruses, adenoviruses, parvovirus B19, cytomegalovirus (CMV), and Epstein-Barr virus (EBV)^[2]^. Recommended therapies for acute severe ulcerative colitis (ASUC) include corticosteroids, biologic agents, and JAK inhibitors; however, these treatments may increase the risk of opportunistic viral infections^[3]^. Viral myocarditis may result in dilated cardiomyopathy, heart failure, and cardiogenic shock^[4]^. The underlying cause determines the treatment for myocarditis, which remains primarily supportive^[4]^.

Increasing studies have shown that gut microbiota play a significant role in the pathogenesis of myocarditis^[5–6]^. Fecal microbiota transplantation (FMT) has been shown to reduce myocardial damage and inflammatory infiltration in mice with autoimmune myocarditis by restoring microbiota composition^[7]^. A new method of FMT, based on an automatic washing process, is called washed microbiota transplantation (WMT), which improves transplantation-related safety and standardizes quantitative methods, and delivery of microbiota suspension^[8–9]^. Here, we report a patient with ASUC complicated by viral myocarditis who rapidly achieved clinical remission through WMT.

The current study was approved by the Institutional Review Board of the Second Affiliated Hospital of Nanjing Medical University (Approval No. [2012]-ky-015). Informed consent was obtained from the patient.

## Case report

A 36-year-old man, who presented with hematochezia, diarrhea, and abdominal pain, was hospitalized on May 7, 2022. Upon admission, he reported more than 10 bloody stools per day, accompanied by anorexia and fatigue. He had been diagnosed with ulcerative colitis (UC) two years prior and had taken mesalazine irregularly since then. He had stopped taking mesalazine six months earlier because of poor compliance. There was no personal or family history of cardiovascular disease.

**Figure 1:**
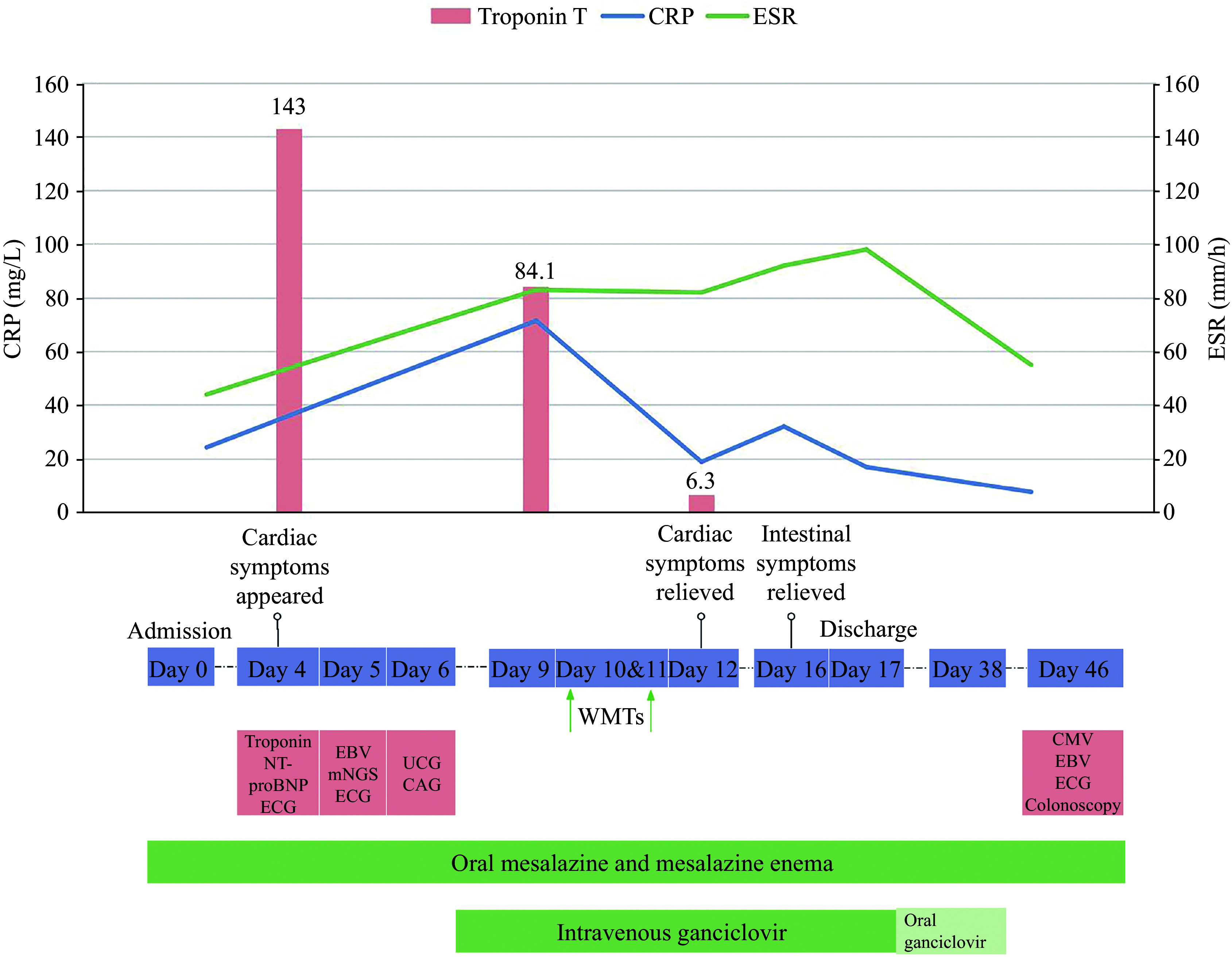
Clinical course and changes of the important laboratory indicators of the patient. Abbreviations: ECG, electrocardiogram; EBV, Epstein-Barr virus; mNGS, metagenomics next-generation sequencing; UCG, ultrasonic cardiogram; CAG, coronary angiography; WMT, washed microbiota transplantation; CMV, cytomegalovirus; CRP, C-reactive protein; ESR, erythrocyte sedimentation rate.

**Figure 2:**
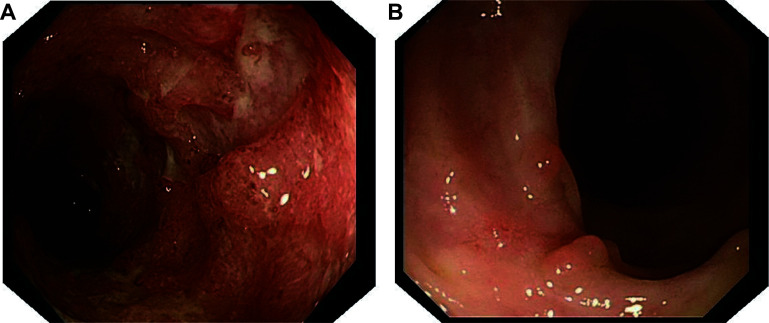
Endoscopy images of the patient before and after WMT. A: Endoscopic findings showed punched-out ulcers before WMT, graded Mayo 3, UCEIS 7. B: Endoscopic findings showed significant improvement without punched-out ulcers one month after WMT, graded Mayo 1, UCEIS 1. Abbreviations: WMT, washed microbiota transplantation; UCEIS, Ulcerative Colitis Endoscopic Index of Severity.

The vital signs on admission were stable (temperature [T], 36.6 ℃; pulse [P], 68/min; respiration [R], 18/min; blood pressure [BP], 124/85 mmHg). Physical examination showed no significant signs except for lower abdominal tenderness and hyperactive bowel sounds. C-reactive protein (CRP) and erythrocyte sedimentation rate (ESR) were 24.3 mg/L and 44 mm/h, respectively (***Fig. 1***). Serological tests revealed positive CMV-IgG (3.63 AU/L). Colonoscopy showed completely absent vessels, significant congestion, erosion, and scattered punched-out ulcers throughout the colon, graded as Mayo 3, with an Ulcerative Colitis Endoscopic Index of Severity (UCEIS) of seven^[10]^ (***Fig. 2A***).

**Figure 3:**
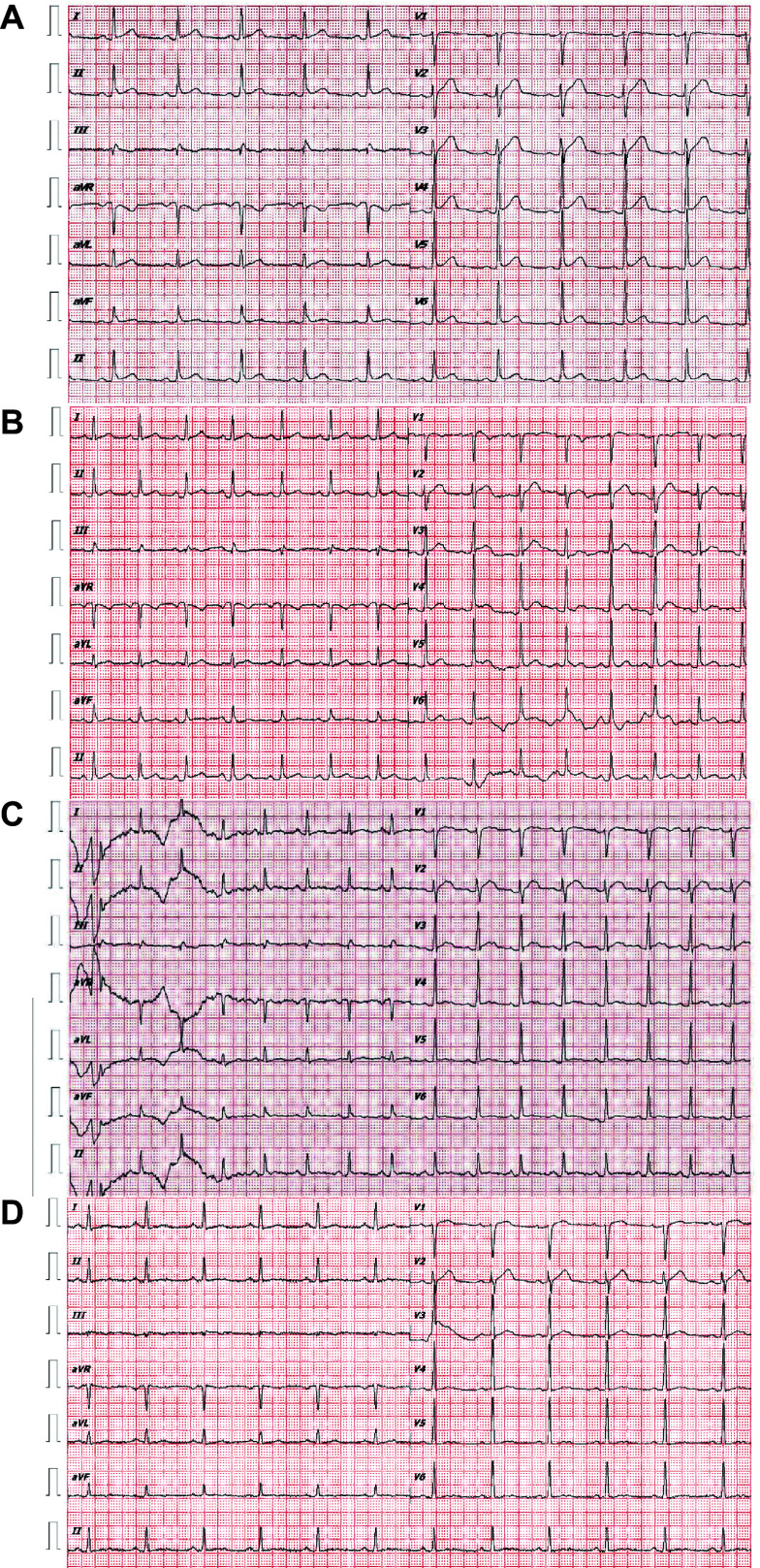
Dynamic ECG changes during the disease. A: The ECG revealed oblique ST-segment elevation in leads Ⅱ and V4 to V6 on May 7. B: Normal ECG on May 11. C: The ECG showed that the T-wave in leads Ⅱ and V4 to V6 changed from upright to flat on May 12. D: The ECG revealed T-wave changes in leads Ⅱ and V4 to V6 on May 17. Abbreviation: ECG, electrocardiogram.

On day 4, the patient experienced persistent chest tightness, with decreased BP (106/65 mmHg) and an elevated heart rate (99/min). Elevated levels of troponin T (143 ng/L), troponin I (0.07 ng/mL), and NT-proBNP (3989 pg/mL) were detected. The electrocardiogram showed dynamic ST-T changes on day 5 (***Fig. 3***). Echocardiography conducted on day 6 revealed normal findings. Myocarditis was highly suspected based on cardiac symptoms, dynamic electrocardiogram changes, and elevated biomarkers of myocardial injury.

To identify the cause of myocardial injury, viral serology for EBV and metagenomics next-generation sequencing (mNGS) were conducted. An endomyocardial biopsy was not performed to confirm the etiological diagnosis of viral myocarditis because of the invasive nature of the procedure. The serological test revealed positive VCA-IgG (45.05 AU/mL) and EBNA-IgG (> 50.00 AU/mL). CMV and EBV were also detected in the blood through mNGS. Coronary angiography was conducted on day 6 to exclude acute coronary artery disease, and no abnormalities were found.

The patient was diagnosed with ASUC complicated by viral myocarditis. Ganciclovir was initiated on day 7 at a dose of 5 mg/kg intravenously, twice daily, for 10 days. Following discharge, treatment was transitioned to oral ganciclovir capsules at a dose of 250 mg three times daily for an additional 21 days. The patient's disease was progressive with conventional treatments, including oral mesalazine and mesalazine enema, as CRP (71.5 mg/L) and ESR (83 mm/h) on day 9 were higher than at admission. Rescue WMT was administered on days 10 and 11 through the colonic transendoscopic enteral tubing after obtaining consent from the patient and his relatives. The symptom of chest tightness disappeared, with stable vital signs (T, 36.6 ℃; P, 70/min; R, 18/min; BP, 114/70 mmHg) on the second day after the initial WMT. The level of troponin T was 6.3 ng/L on the same day. From the sixth day post-WMT, the patient had yellow loose stools once daily without abdominal pain. The patient was discharged seven days post-WMT. CRP and serological tests for CMV and EBV normalized one month after WMT. A colonoscopy conducted one month after WMT showed a small number of aphthous ulcers in the rectum and sigmoid, as well as scars in the mucosa of the whole colon, graded as Mayo 1, with an UCEIS of one (***Fig. 2B***). During the one-year follow-up, UC relapsed once nine months post-WMT, but clinical remission was achieved after the second course of WMT, with no recurrent cardiac symptoms. No adverse events were observed during the short-term and long-term follow-up after WMT.

## Discussion

In this case, WMT effectively treated ASUC complicated by viral myocarditis. The patient's clinical manifestations and examination results met the diagnostic criteria for myocarditis^[11]^. Both CMV and EBV are cardiotropic viruses that may lead to viral myocarditis with a poor prognosis^[4]^. The etiology of myocarditis in this case was attributed to CMV/EBV co-infection, as supported by seropositivity for both viruses and detection by mNGS. Mesalazine toxicity and UC-related extra-intestinal manifestations were excluded because of the six-month treatment discontinuation and the absence of prior cardiac symptoms.

Myocardial injury in viral myocarditis results from direct damage mediated by viral infection and secondary immune responses. Gut microbiota reconstruction may inhibit CMV infection by regulating the activation of CD8^+^ T cells and increasing the expression of type Ⅰ interferon signaling pathways^[12]^. Furthermore, microbiota-derived short-chain fatty acids (SCFAs) may enhance T helper cell function and maintain gut epithelial barrier integrity, which helps prevent aberrant inflammatory responses^[13]^. Tang *et al*^[14]^ found that FMT could promote cardiac repair in mice with myocardial infarction by increasing the abundance of SCFA-producing *Lactobacillus*. In this case, the patient's intestinal and cardiac symptoms improved after WMT, an effective method for reconstructing gut microbiota. The mechanisms by which WMT treats viral myocarditis may involve increasing the abundance of SCFA-producing bacteria and supporting the immune system.

Patients with inflammatory bowel disease infected with EBV or CMV may experience a prolonged course of the disease, serious complications, and frequent recurrences^[15]^. WMT was administered three days after the commencement of antiviral therapy in response to disease progression. The patient's intestinal symptoms improved significantly on the sixth day after WMT. Eighteen days after the end of antiviral therapy, which was much shorter than the three months reported in Rachele Ciccocioppo's study^[16]^, the colonoscopy revealed the disappearance of characteristic ulcers and old scars in the mucosa of the entire colon, with a reduction in the UCEIS score from seven to one. These findings suggest that WMT accelerated clinical and endoscopic remission in ASUC with CMV and EBV infections.

The treatment of UC with viral infections is complex because the use of corticosteroids or immunosuppressants may exacerbate the infection. Increasing studies have demonstrated the efficacy of FMT in UC^[17]^ and the safety of FMT in immunocompromised populations^[18]^. Zhang *et al*^[19]^ reported that eukaryotic viruses were reduced after FMT in a patient with graft-versus-host disease, indicating the potential of FMT in eliminating pathogenic viruses. WMT is a newer generation of FMT that reduces the incidence of adverse events in patients with UC from 35.5% to 7.2%^[20]^. Given the favorable safety of WMT and the established association between gut microbiota and viral infections, we applied the rescue WMT, and the patient's intestinal and cardiac symptoms rapidly improved without any adverse events.

In conclusion, this case demonstrates that a patient with ASUC complicated by viral myocarditis was successfully treated with WMT, suggesting a new potential treatment option for these life-threatening conditions.
